# 1-Methyl­hydrazinium picrate

**DOI:** 10.1107/S1600536811048677

**Published:** 2011-11-19

**Authors:** Xiao-Gang Mu, Xuan-Jun Wang, Youzhi Zhang, Xiaoli Gou, Xia Li

**Affiliations:** a No. 503 Faculty, Xi’an Research Institute of High Technology, Hongqing Town, Xi’an 710025, People’s Republic of China

## Abstract

In the title salt, CH_7_N_2_^+^·C_6_H_2_N_3_O_7_^−^, the dihedral angles between the three nitro groups and the plane of the benzene ring are 22.4 (2), 35.3 (2) and 2.8 (2)°. In the crystal, the components are linked by N—H⋯O and N—H⋯N hydrogen bonds into a two-dimensional network parallel to (10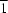).

## Related literature

For related structures, see: Yang *et al.* (2002 ▶); Mu *et al.* (2011 ▶).
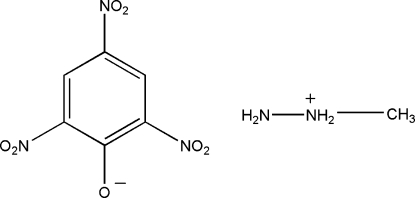


## Experimental

### 

#### Crystal data

CH_7_N_2_^+^·C_6_H_2_N_3_O_7_^−^
*M* *_r_* = 275.19
Monoclinic, 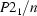

*a* = 11.766 (3) Å
*b* = 6.785 (2) Å
*c* = 14.420 (4) Å
β = 110.526 (4)°
*V* = 1078.0 (5) Å^3^
*Z* = 4
Mo *K*α radiation
μ = 0.15 mm^−1^
*T* = 296 K
0.33 × 0.25 × 0.14 mm

#### Data collection

Bruker APEXII diffractometer
Absorption correction: multi-scan (*SADABS*; Sheldrick, 1996 ▶) *T*_min_ = 0.952, *T*_max_ = 0.979
5196 measured reflections
1907 independent reflections
1562 reflections with *I* > 2σ(*I*)
*R*_int_ = 0.023

#### Refinement

*R*[*F* ^2^ > 2σ(*F* ^2^)] = 0.037
*wR*(*F* ^2^) = 0.103
*S* = 1.08
1907 reflections
173 parameters
H-atom parameters constrained
Δρ_max_ = 0.15 e Å^−3^
Δρ_min_ = −0.26 e Å^−3^

### 

Data collection: *APEX2* (Bruker, 2007 ▶); cell refinement: *SAINT* (Bruker, 2007 ▶); data reduction: *SAINT*; program(s) used to solve structure: *SHELXS97* (Sheldrick, 2008 ▶); program(s) used to refine structure: *SHELXL97* (Sheldrick, 2008 ▶); molecular graphics: *PLATON* (Spek, 2009 ▶); software used to prepare material for publication: *SHELXTL* (Sheldrick, 2008 ▶).

## Supplementary Material

Crystal structure: contains datablock(s) global, I. DOI: 10.1107/S1600536811048677/lh5375sup1.cif

Structure factors: contains datablock(s) I. DOI: 10.1107/S1600536811048677/lh5375Isup2.hkl

Supplementary material file. DOI: 10.1107/S1600536811048677/lh5375Isup3.cml

Additional supplementary materials:  crystallographic information; 3D view; checkCIF report

## Figures and Tables

**Table 1:** Hydrogen-bond geometry (Å, °)

*D*—H⋯*A*	*D*—H	H⋯*A*	*D*⋯*A*	*D*—H⋯*A*
N4—H4*A*⋯O3^i^	0.88	2.03	2.7807 (19)	142
N4—H4*A*⋯O2^i^	0.88	2.25	2.963 (2)	138
N4—H4*B*⋯N5^ii^	0.85	2.13	2.954 (2)	161
N5—H5*A*⋯O3^iii^	0.87	2.36	3.156 (2)	151
N5—H5*B*⋯O4^iv^	0.90	2.59	3.377 (2)	146

Symmetry codes: (i) 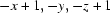; (ii) 
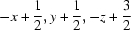
; (iii) 
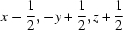
; (iv) 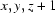.

**Fig. 1.**
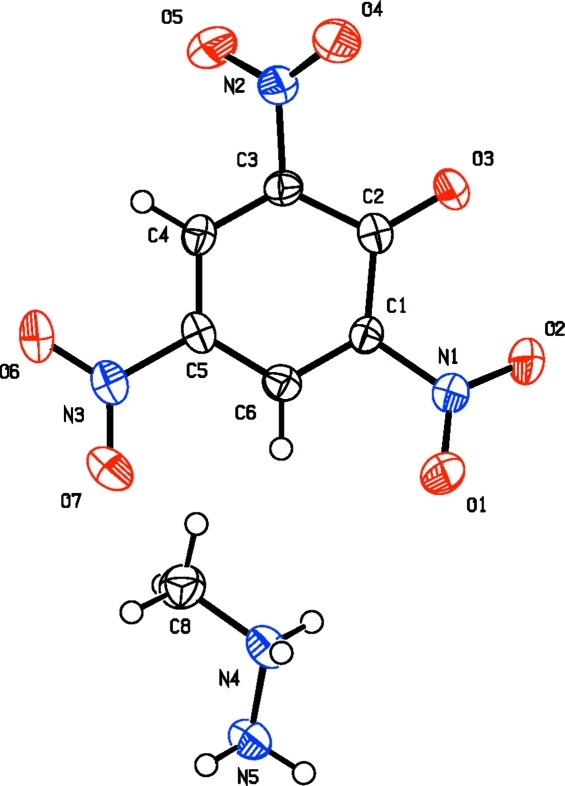
The molecular structure of the title compound showing 30% probability displacement ellipsoids.

## supplementary crystallographic information

### Comment

The molecular structure of the title compound is shown in Fig. 1. The dihedral angles between the three nitro groups and the plane of the benzene ring are 22.4 (2), 35.3 (2) and 2.8 (2)° for the groups containing N1, N2 and N3. In the crystal, the components are linked by N—H···O hydrogen bonds into a two-dimensional network paralell to (101).

### Experimental

1-methylhydrazine (0.02 mol) was added to a solution of picric acid (0.02 mol) in 30 ml ethanol at room temperature, the mixture was stirred for 0.6 h to afford the title compound. Single crystals suitable for X-ray structural analysis was obtained by slowly evaporating from distilled water at room temperature.

### Refinement

H atoms were fixed geometrically and allowed to ride on their attached atoms, with C—H distances = 0.93–0.96 Å; N—H = 0.85-0.90 Å and with *U*_iso_(H) = 1.2*U*_eq_(C,*N*) or 1.5*U*_eq_(C_methyl_).

### Crystal data

**Table:**

CH_7_N_2_^+^·C_6_H_2_N_3_O_7_^−^	*F*(000) = 568
*M**_r_* = 275.19	*D*_x_ = 1.696 Mg m^−^^3^
Monoclinic, *P*2_1_/*n*	Mo *K*α radiation, λ = 0.71073 Å
Hall symbol: -P 2yn	Cell parameters from 1614 reflections
*a* = 11.766 (3) Å	θ = 2.8–26.1°
*b* = 6.785 (2) Å	µ = 0.15 mm^−^^1^
*c* = 14.420 (4) Å	*T* = 296 K
β = 110.526 (4)°	Block, yellow
*V* = 1078.0 (5) Å^3^	0.33 × 0.25 × 0.14 mm
*Z* = 4	

### Data collection

**Table:**

Bruker APEXII diffractometer	1907 independent reflections
Radiation source: fine-focus sealed tube	1562 reflections with *I* > 2σ(*I*)
graphite	*R*_int_ = 0.023
φ and ω scans	θ_max_ = 25.1°, θ_min_ = 1.9°
Absorption correction: multi-scan (*SADABS*; Sheldrick, 1996)	*h* = −12→14
*T*_min_ = 0.952, *T*_max_ = 0.979	*k* = −8→7
5196 measured reflections	*l* = −17→12

### Refinement

**Table:**

Refinement on *F*^2^	Primary atom site location: structure-invariant direct methods
Least-squares matrix: full	Secondary atom site location: difference Fourier map
*R*[*F*^2^ > 2σ(*F*^2^)] = 0.037	Hydrogen site location: inferred from neighbouring sites
*wR*(*F*^2^) = 0.103	H-atom parameters constrained
*S* = 1.08	*w* = 1/[σ^2^(*F*_o_^2^) + (0.0537*P*)^2^ + 0.153*P*] where *P* = (*F*_o_^2^ + 2*F*_c_^2^)/3
1907 reflections	(Δ/σ)_max_ < 0.001
173 parameters	Δρ_max_ = 0.15 e Å^−^^3^
0 restraints	Δρ_min_ = −0.26 e Å^−^^3^

### Special details

**Table:**

Geometry. All e.s.d.'s (except the e.s.d. in the dihedral angle between two l.s. planes) are estimated using the full covariance matrix. The cell e.s.d.'s are taken into account individually in the estimation of e.s.d.'s in distances, angles and torsion angles; correlations between e.s.d.'s in cell parameters are only used when they are defined by crystal symmetry. An approximate (isotropic) treatment of cell e.s.d.'s is used for estimating e.s.d.'s involving l.s. planes.
Refinement. Refinement of *F*^2^ against ALL reflections. The weighted *R*-factor *wR* and goodness of fit *S* are based on *F*^2^, conventional *R*-factors *R* are based on *F*, with *F* set to zero for negative *F*^2^. The threshold expression of *F*^2^ > σ(*F*^2^) is used only for calculating *R*-factors(gt) *etc*. and is not relevant to the choice of reflections for refinement. *R*-factors based on *F*^2^ are statistically about twice as large as those based on *F*, and *R*- factors based on ALL data will be even larger.

### Fractional atomic coordinates and isotropic or equivalent isotropic displacement parameters (Å^2^)

**Table:**

	*x*	*y*	*z*	*U*_iso_*/*U*_eq_	
N1	0.52167 (12)	0.1473 (2)	0.41558 (10)	0.0358 (4)	
N2	0.31795 (13)	0.0736 (2)	0.04968 (10)	0.0359 (4)	
N3	0.08521 (12)	0.1090 (2)	0.26709 (11)	0.0353 (4)	
N4	0.26648 (12)	0.1526 (2)	0.69636 (10)	0.0335 (4)	
H4A	0.3278	0.0814	0.6943	0.040*	
H4B	0.2808	0.2764	0.7014	0.040*	
N5	0.24219 (13)	0.0849 (2)	0.78250 (11)	0.0361 (4)	
H5A	0.1824	0.1576	0.7857	0.043*	
H5B	0.3089	0.1102	0.8361	0.043*	
O1	0.50471 (11)	0.2240 (2)	0.48615 (9)	0.0523 (4)	
O2	0.62224 (11)	0.0934 (3)	0.41872 (10)	0.0575 (4)	
O3	0.53492 (10)	0.09419 (19)	0.22096 (8)	0.0365 (3)	
O4	0.39264 (12)	0.1642 (3)	0.02613 (10)	0.0612 (5)	
O5	0.23853 (12)	−0.0268 (2)	−0.00860 (9)	0.0536 (4)	
O6	−0.00559 (11)	0.0933 (2)	0.19306 (11)	0.0578 (4)	
O7	0.08063 (12)	0.1317 (2)	0.34954 (11)	0.0568 (4)	
C1	0.41718 (13)	0.1197 (2)	0.32599 (11)	0.0268 (4)	
C2	0.43426 (14)	0.1013 (2)	0.23195 (12)	0.0266 (4)	
C3	0.31911 (14)	0.0895 (2)	0.15092 (11)	0.0275 (4)	
C4	0.20832 (14)	0.0859 (2)	0.16195 (12)	0.0281 (4)	
H4	0.1377	0.0719	0.1070	0.034*	
C5	0.20249 (14)	0.1034 (2)	0.25554 (12)	0.0274 (4)	
C6	0.30624 (14)	0.1220 (2)	0.33767 (12)	0.0285 (4)	
H6	0.3013	0.1359	0.4003	0.034*	
C8	0.16207 (17)	0.1100 (3)	0.60533 (14)	0.0435 (5)	
H8A	0.1426	−0.0278	0.6032	0.065*	
H8B	0.1825	0.1437	0.5484	0.065*	
H8C	0.0933	0.1862	0.6052	0.065*	

### Atomic displacement parameters (Å^2^)

**Table:**

	*U*^11^	*U*^22^	*U*^33^	*U*^12^	*U*^13^	*U*^23^
N1	0.0281 (8)	0.0469 (10)	0.0311 (8)	−0.0041 (6)	0.0089 (6)	−0.0046 (7)
N2	0.0319 (8)	0.0462 (9)	0.0298 (8)	0.0034 (7)	0.0111 (7)	0.0037 (7)
N3	0.0277 (8)	0.0379 (9)	0.0427 (9)	−0.0019 (6)	0.0155 (7)	0.0004 (6)
N4	0.0337 (8)	0.0286 (8)	0.0433 (9)	0.0012 (6)	0.0200 (7)	0.0013 (6)
N5	0.0365 (8)	0.0383 (9)	0.0383 (8)	0.0033 (6)	0.0192 (7)	0.0011 (6)
O1	0.0407 (7)	0.0773 (11)	0.0365 (7)	−0.0070 (7)	0.0104 (6)	−0.0241 (7)
O2	0.0246 (7)	0.1020 (13)	0.0408 (8)	0.0096 (7)	0.0051 (6)	−0.0088 (8)
O3	0.0244 (6)	0.0512 (8)	0.0367 (7)	0.0023 (5)	0.0142 (5)	0.0039 (5)
O4	0.0478 (8)	0.1007 (13)	0.0388 (8)	−0.0176 (8)	0.0196 (7)	0.0114 (8)
O5	0.0514 (8)	0.0733 (11)	0.0330 (7)	−0.0126 (8)	0.0111 (6)	−0.0139 (7)
O6	0.0231 (7)	0.0945 (12)	0.0524 (9)	−0.0012 (7)	0.0092 (6)	−0.0003 (8)
O7	0.0425 (8)	0.0867 (12)	0.0515 (9)	−0.0072 (7)	0.0294 (7)	−0.0082 (8)
C1	0.0229 (8)	0.0294 (9)	0.0260 (8)	−0.0015 (6)	0.0057 (6)	−0.0015 (6)
C2	0.0249 (8)	0.0224 (9)	0.0329 (9)	0.0000 (6)	0.0105 (7)	0.0015 (6)
C3	0.0301 (9)	0.0281 (9)	0.0243 (8)	0.0009 (7)	0.0094 (7)	0.0028 (6)
C4	0.0235 (8)	0.0279 (9)	0.0294 (8)	0.0006 (6)	0.0050 (7)	0.0028 (7)
C5	0.0228 (8)	0.0253 (9)	0.0361 (9)	0.0008 (6)	0.0127 (7)	0.0018 (7)
C6	0.0304 (9)	0.0277 (9)	0.0291 (8)	−0.0002 (7)	0.0126 (7)	−0.0013 (7)
C8	0.0428 (11)	0.0478 (12)	0.0381 (10)	0.0078 (9)	0.0118 (9)	−0.0004 (8)

### Geometric parameters (Å, °)

**Table:**

N1—O1	1.2200 (18)	N5—H5B	0.9046
N1—O2	1.2240 (18)	O3—C2	1.2498 (19)
N1—C1	1.450 (2)	C1—C6	1.374 (2)
N2—O4	1.2143 (19)	C1—C2	1.444 (2)
N2—O5	1.2216 (19)	C2—C3	1.448 (2)
N2—C3	1.459 (2)	C3—C4	1.368 (2)
N3—O7	1.2186 (19)	C4—C5	1.380 (2)
N3—O6	1.2217 (19)	C4—H4	0.9300
N3—C5	1.447 (2)	C5—C6	1.376 (2)
N4—N5	1.4439 (19)	C6—H6	0.9300
N4—C8	1.478 (2)	C8—H8A	0.9600
N4—H4A	0.8777	C8—H8B	0.9600
N4—H4B	0.8546	C8—H8C	0.9600
N5—H5A	0.8732		
			
O1—N1—O2	122.36 (14)	O3—C2—C1	124.91 (14)
O1—N1—C1	117.56 (14)	O3—C2—C3	123.77 (15)
O2—N1—C1	120.08 (14)	C1—C2—C3	111.31 (13)
O4—N2—O5	122.94 (15)	C4—C3—C2	124.57 (15)
O4—N2—C3	119.25 (15)	C4—C3—N2	116.12 (14)
O5—N2—C3	117.77 (14)	C2—C3—N2	119.28 (14)
O7—N3—O6	122.63 (15)	C3—C4—C5	119.24 (15)
O7—N3—C5	119.05 (14)	C3—C4—H4	120.4
O6—N3—C5	118.31 (15)	C5—C4—H4	120.4
N5—N4—C8	110.36 (14)	C6—C5—C4	121.05 (14)
N5—N4—H4A	105.5	C6—C5—N3	119.50 (15)
C8—N4—H4A	107.4	C4—C5—N3	119.41 (14)
N5—N4—H4B	109.4	C1—C6—C5	119.20 (15)
C8—N4—H4B	110.2	C1—C6—H6	120.4
H4A—N4—H4B	113.8	C5—C6—H6	120.4
N4—N5—H5A	105.7	N4—C8—H8A	109.5
N4—N5—H5B	107.5	N4—C8—H8B	109.5
H5A—N5—H5B	108.8	H8A—C8—H8B	109.5
C6—C1—C2	124.55 (14)	N4—C8—H8C	109.5
C6—C1—N1	115.77 (14)	H8A—C8—H8C	109.5
C2—C1—N1	119.61 (14)	H8B—C8—H8C	109.5
			
O1—N1—C1—C6	−20.3 (2)	O4—N2—C3—C2	−37.3 (2)
O2—N1—C1—C6	159.30 (16)	O5—N2—C3—C2	144.91 (16)
O1—N1—C1—C2	156.85 (16)	C2—C3—C4—C5	3.1 (3)
O2—N1—C1—C2	−23.5 (2)	N2—C3—C4—C5	−178.99 (14)
C6—C1—C2—O3	−178.15 (16)	C3—C4—C5—C6	−0.8 (2)
N1—C1—C2—O3	4.9 (2)	C3—C4—C5—N3	177.12 (15)
C6—C1—C2—C3	1.2 (2)	O7—N3—C5—C6	0.6 (2)
N1—C1—C2—C3	−175.74 (14)	O6—N3—C5—C6	179.60 (15)
O3—C2—C3—C4	176.18 (16)	O7—N3—C5—C4	−177.37 (15)
C1—C2—C3—C4	−3.1 (2)	O6—N3—C5—C4	1.7 (2)
O3—C2—C3—N2	−1.7 (2)	C2—C1—C6—C5	0.8 (2)
C1—C2—C3—N2	178.99 (14)	N1—C1—C6—C5	177.81 (14)
O4—N2—C3—C4	144.66 (17)	C4—C5—C6—C1	−1.1 (2)
O5—N2—C3—C4	−33.1 (2)	N3—C5—C6—C1	−178.97 (15)

### Hydrogen-bond geometry (Å, °)

**Table:**

*D*—H···*A*	*D*—H	H···*A*	*D*···*A*	*D*—H···*A*
N4—H4A···O3^i^	0.88	2.03	2.7807 (19)	142.
N4—H4A···O2^i^	0.88	2.25	2.963 (2)	138.
N4—H4B···N5^ii^	0.85	2.13	2.954 (2)	161.
N5—H5A···O3^iii^	0.87	2.36	3.156 (2)	151.
N5—H5B···O4^iv^	0.90	2.59	3.377 (2)	146.

Symmetry codes: (i) −*x*+1, −*y*, −*z*+1; (ii) −*x*+1/2, *y*+1/2, −*z*+3/2; (iii) *x*−1/2, −*y*+1/2, *z*+1/2; (iv) *x*, *y*, *z*+1.
